# Blood-brain barrier injury and neuroinflammation in pre-eclampsia and eclampsia

**DOI:** 10.1016/j.ebiom.2025.105742

**Published:** 2025-05-08

**Authors:** Valentina Bucher, Owen T. Herrock, Sonja Schell, Jacqui Visser, Henrik Imberg, Jonathan Burke, Henrik Zetterberg, Kaj Blennow, Susan P. Walker, Stephen Tong, Joakim Ek, Catherine Cluver, Lina Bergman

**Affiliations:** a Department of Obstetrics and Gynecology, Institute of Clinical Sciences, Sahlgrenska Academy, University of Gothenburg, Gothenburg, Sweden; b Department of Obstetrics and Gynecology, Stellenbosch University, Tygerberg Hospital, Cape Town, South Africa; c Statistiska Konsultgruppen Sweden, Gothenburg, Sweden; d Department of Molecular and Clinical Medicine, Institute of Medicine, Sahlgrenska Academy, University of Gothenburg, Gothenburg, Sweden; e Department of Anaesthesia and Critical Care, Faculty of Medicine and Health Sciences, Stellenbosch University, Tygerberg Hospital, Cape Town, South Africa; f Department of Psychiatry and Neurochemistry, Institute of Neuroscience and Physiology, Sahlgrenska, Academy, University of Gothenburg, Mölndal, Sweden; g Clinical Neurochemistry Laboratory, Sahlgrenska University Hospital, Mölndal, Sweden; h Department of Neurodegenerative Disease, UCL Institute of Neurology, London, UK; i UK Dementia Research Institute, London, UK; j Hong Kong Center for Neurodegenerative Diseases, Hong Kong, China; k Wisconsin Alzheimer's Disease Research Center, University of Wisconsin School of Medicine and Public Health, Madison, WI, USA; l Translational Obstetrics Group, Department of Obstetrics and Gynaecology, University of Melbourne, Victoria, Australia; m Mercy Perinatal, Mercy Hospital for Women, Heidelberg, Victoria, Australia; n Institute of Neuroscience and Physiology, Sahlgrenska Academy, University of Gothenburg, Gothenburg, Sweden; o Department of Obstetrics and Gynecology, Region Västra Götaland, Sahlgrenska, University Hospital, Goteborg, Sweden; p Department of Women's and Children's Health, Uppsala University, Uppsala, Sweden

**Keywords:** Pre-eclampsia, Eclampsia, Neuroinflammation, Blood-brain barrier, Maternal complications

## Abstract

**Background:**

Cerebral complications of pre-eclampsia are a leading cause of maternal mortality. Better understanding of the pathophysiology may enable the development of novel strategies to protect the maternal brain. We aimed to investigate blood-brain barrier injury and neuroinflammatory pathways in women with eclampsia and pre-eclampsia compared to normotensive pregnancies.

**Methods:**

This observational cross-sectional study conducted between March 2021 and June 2023, included women with eclampsia, pre-eclampsia, and normotensive pregnancies admitted to Tygerberg Hospital, Cape Town, South Africa who underwent caesarean delivery. Cerebrospinal fluid and plasma samples were collected during caesarean delivery. Blood-brain barrier injury was assessed using immunonephelometry for albumin and ELISA assays for claudin-5 and matrix metalloproteinase-9 (MMP-9). Neuroinflammatory markers were analysed on the multiplex Bio-Plex Pro Human Cytokine-Screening assay. Data were analysed using parametric methods after log transformation and are presented as fold changes (geometric mean ratios) between groups.

**Findings:**

The study included 129 women: Eleven had eclampsia, 17 had pre-eclampsia with end-organ complications, 88 had pre-eclampsia without end-organ complications, and 13 with normotensive pregnancies. Women with eclampsia had increased cerebrospinal fluid concentrations of claudin-5 (2.7-fold, 95% CI 1.4–5.1, p = 0.002 vs normotensive control) and MMP-9 (2.5-fold, 95% CI 1.1–5.3, p = 0.024 vs pre-eclampsia with end-organ complications). They also demonstrated increased cerebrospinal fluid cytokine levels compared to normotensive controls, reflecting inflammatory recruitment (Interleukin-8: 7.2-fold, 95% CI 2.7–18.5, p < 0.001), cytotoxicity (Interleukin-6: 20.7-fold, 95% CI 6.4–63.6, p < 0.001), and immune modulation (Interleukin-10: 2.0-fold, 95% CI 1.2–3.1, p = 0.004). Neuroprotective markers were reduced in eclampsia (stem cell factor: 0.5-fold, 95% CI 0.3–0.8, p = 0.005) compared to normotensive controls. There was no correlation between cytokine concentrations in the cerebrospinal fluid and plasma. Women with pre-eclampsia showed less pronounced changes indicative of blood-brain barrier injury and immune modulation.

**Interpretation:**

Eclampsia is associated with blood-brain barrier injury and acute neuroinflammation originating from cerebral tissue, inducing cytotoxicity. This may be an underlying mechanism for seizures and cerebral injury in eclampsia and pre-eclampsia.

**Funding:**

This study was supported by the 10.13039/501100004359Swedish Research Council, Herbert och Karin Jacobsson Stiftelse, 10.13039/501100003745Wilhelm and Martina Lundgren Foundations, and 10.13039/501100007687Swedish Society Of Medicine.

**Research in context**

**Evidence before this study**

Two studies of pre-eclampsia, without cases of eclampsia, reported no differences in blood-brain barrier injury. In a small case-series, our group found evidence of blood-brain barrier injury and neuroinflammation in pre-eclampsia and eclampsia. However, the pathophysiological mechanisms remain poorly studied.

We searched the databases Medline, Embase, CINAHL, and Cochrane library and performed citation tracking from the previous articles until January 2025, to identify if there were new articles using the terms including: “Blood-brain barrier injury”, “Claudin-5”, “Cerebral complications”, “Neuroinflammation”, “Pre-eclampsia”, “Eclampsia” without any language or time restriction and did not identify any other study with analysis of clinical cerebrospinal fluid samples of women with eclampsia.

**Added value of this study**

This observational study revealed signs of blood-brain barrier injury in women with eclampsia, indicated by tight-junction protein degradation. Those women suffer from acute neuroinflammation, reflected by increased levels of inflammatory recruitment, cytotoxic, and immune-modulating markers in cerebrospinal fluid. The levels of inflammatory markers in cerebrospinal fluid did not correlate with levels in plasma which indicates that the neuroinflammation originates from the cerebral tissue and not through leakage of systemic inflammatory markers through the blood-brain barrier.

**Implications of all the available evidence**

Eclampsia and pre-eclampsia are associated with blood-brain barrier injury and a subsequent acute neuroinflammatory response, originating from the cerebral tissue. These pathophysiological changes resemble those in neurodegenerative diseases such as Alzheimer's disease and traumatic brain injury. Clinical surveillance and long-term follow-up of women with eclampsia should be considered to identify persisting neurological sequelae and provide appropriate support.

## Introduction

Pre-eclampsia is a multi-organ endothelial disorder of pregnancy,^1^ complicating 5% of pregnancies and causing over 60,000 maternal deaths globally each year.^2^

Cerebral complications of pre-eclampsia, such as eclampsia, cerebral oedema, and intracranial haemorrhage, are major contributors to direct maternal mortality.^3^^,^^4^ Women who experience pre-eclampsia and associated complications face an increased risk for stroke, dementia, migraine, epilepsy, and cognitive impairment later in life.^5^, ^6^, ^7^, ^8^, ^9^ Improved understanding of the pathophysiology may allow for better detection and the development of novel interventions to prevent and treat cerebral complications and long-term consequences.

Impaired cerebral blood flow autoregulation, blood-brain barrier injury, and neuroinflammation are proposed mechanisms underlying cerebral complications.^3^^,^^10^, ^11^, ^12^ Animal models of pre-eclampsia and *in vitro* studies have demonstrated blood-brain barrier injury and increased neuroinflammation,^13^, ^14^, ^15^, ^16^, ^17^, ^18^ though clinical data remain limited. An observational study of women with pre-eclampsia, but without eclampsia, found no evidence of blood-brain barrier injury or neuroinflammatory changes in cerebrospinal fluid.^19^ In a small case-series, our group found evidence of blood-brain barrier injury and neuroinflammation in pre-eclampsia and eclampsia, but the sample size was very small and restricted by a limited number of markers.^20^ Pathophysiological mechanisms and the origin of neuroinflammation remain unknown.

To address these gaps, we conducted a larger study with comprehensive analyses to examine blood-brain barrier injury and neuroinflammation in women with eclampsia, pre-eclampsia, and normotensive pregnancies.

## Methods

### Study design

In this observational study, we included women who were recruited to the Pre-eclampsia Obstetric Adverse Events (PROVE) biobank at Tygerberg Hospital, a tertiary referral hospital in Cape Town, South Africa, and who had plasma and/or cerebrospinal fluid samples collected at caesarean section between March 2021 and June 2023.^21^

Informed consent was obtained. Demographics and outcome data were collected by research staff, and entered into a REDCap database, hosted by Stellenbosch University.^22^ All data was checked for accuracy.

Women with singleton pregnancies giving birth by caesarean section where cerebrospinal fluid could be collected were included. Exclusion criteria were preexisting neurological or cardiac disease.^21^

### Ethics

Ethics approval was obtained from the Stellenbosch University Health Research Ethics Committee (Project ID 25532 N22/06/063_Sub Study N17/05/048) and the Swedish Ethical Review Authority (Dnr 2023-04689-01). Informed consent was obtained.

### Exposures

Main exposures were pre-eclampsia and eclampsia. Pre-eclampsia was defined as a pregnancy complicated by hypertension (systolic blood pressure ≥140 mm Hg and/or diastolic blood pressure ≥90 mm Hg) together with significant proteinuria (≥2+ proteinuria on urine dipsticks and/or protein/creatinine ratio >30 mmol/mol in 24-h urine collection) after 20 gestational weeks. Pre-eclampsia was sub-categorised as disease with, or without end-organ complications according to a modified Delphi consensus.^23^ End-organ complications included pulmonary oedema, haemolysis, elevated liver enzymes, low-platelet-count (HELLP) syndrome, and acute kidney injury (creatinine >120 μmol/L) (Supplementary Table S1).^23^ Eclampsia was defined as the occurrence of generalised tonic-clonic seizures associated with pre-eclampsia where there was no other neurological cause for the seizure.

Controls were normotensive pregnant women with blood pressures <140/90 mm Hg, without preexisting chronic diseases including chronic hypertension.

### Procedures

Blood samples were collected in ethylenediaminetetraacetic acid-tubes during caesarean section. Cerebrospinal fluid was collected after insertion of the spinal needle for anaesthesia performed for the caesarean section. CSF samples were visually inspected and excluded if blood contaminated.

All samples were centrifuged, aliquoted and frozen within 2 h of collection and stored at −70 °C. Frozen samples were shipped to Gothenburg University, Sweden, for analysis.

### Outcomes

Blood-brain barrier injury was evaluated by measuring the cerebrospinal fluid concentrations of dissociated tight junction protein claudin-5, tight junction protein-degrading matrix metalloprotease-9 (MMP-9) and the cerebrospinal fluid to plasma albumin ratio.

Neuroinflammation was assessed by measuring concentrations of interleukin-6 (IL-6), interleukin-8 (IL-8), and tumour-necrosis factor alpha (TNF-α) in cerebrospinal fluid.^20^ Forty-five cytokines, chemokines, and growth factors were also analysed in cerebrospinal fluid as exploratory outcome (Supplementary Table S2).

To identify pathophysiological pathways over the neurovascular unit, we analysed the correlation between blood-brain barrier injury markers and neuroinflammatory markers in cerebrospinal fluid, as well as correlations between cerebrospinal fluid and plasma.

Cytokines, chemokines, and growth factors were measured using the Bio-Plex Multiplex immunoassay (Bio-Plex Pro Human Cytokine-Screening-Panel, 48-Plex #12007283, Bio-Rad Laboratories Inc., Hercules, CA, USA) according to the manufacturer's protocol. Cerebrospinal fluid samples were diluted 2-fold and plasma samples 4-fold and analysed using four 96-well plates. Cytokines were divided into three physiological pathways: recruitment of inflammation, cytotoxic signals, and immune modulation.

Claudin-5 protein and MMP-9 protein concentrations were measured in cerebrospinal fluid using a human claudin-5 ELISA kit (#CSB-EL005507HU, Cusabio Technology LLC, Houston, TX, USA), and a human MMP9 Immunoassay (#DMP900, R&D Systems, Minneapolis, MN, USA) according to the manufacturer's protocol. The samples were diluted 2-fold.

Albumin concentrations in cerebrospinal fluid and plasma were measured using immunonephelometry on a Beckman Immage system (Beckman Instruments, Beckman Coulter, Brea, CA, USA). The ratio of cerebrospinal fluid to plasma albumin was calculated as cerebrospinal fluid albumin (mg/L)/plasma albumin (g/L).

Investigators were blinded for all outcome measurements.

### Statistical analyses

Demographics and baseline characteristics were summarised as mean with standard deviation or median with interquartile range for numeric variables. Categorical variables were summarised as counts and percentages. Outcome variables were log-transformed prior to analysis accounting for right-skewed distributions and were descriptively reported as geometric mean and coefficient of variation (CV), appropriate for strictly positive data. After transformation, approximate normality was obtained.

Inflammatory cytokines and blood-brain barrier markers were analysed using Welch's analysis of covariance (ANCOVA) to account for unequal variances across groups and adjusted for analytic plate effects, maternal age, body mass index, human immunodeficiency virus status, and chronic hypertension. For variables with concentrations below the detection limit, a log-normal Tobit regression model was employed to address censoring.^24^^,^^25^ Values extrapolated outside the assay range were analysed as recorded. Results were reported as fold changes (geometric mean ratios) with 95% confidence intervals.

Exploratory outcomes (Supplementary Table S2) were assessed using an omnibus F-test adjusted for plate effects. Multiple testing corrections were applied using the Benjamini-Hochberg procedure to control the false discovery rate (FDR) at 10%. Biomarkers meeting the significance threshold of a 10% FDR were further evaluated the same ANCOVA model as described above.

Correlation analyses for non-censored data were conducted using Pearson correlation coefficients on log-transformed values. For censored data, correlation coefficients were estimated using log-Gaussian profile likelihood methods implemented in the clikcorr R package.^26^ Correlation coefficients were interpreted as follows: ≤0.3, weak; ≥0.3–0.5, fair; ≥0.5–0.7; and ≥0.7–0.9, high; ≥0.9, very high.^27^

With n = 11 women with eclampsia, n = 13 normotensive controls, n = 17 with pre-eclampsia with end-organ complications, and n = 88 with pre-eclampsia without end-organ complications, the study was powered at 80% to detect large effects when comparing eclampsia to the other groups (effect sizes >1.20 vs normotensive, >1.15 vs severe pre-eclampsia, and >0.90 vs pre-eclampsia without end-organ complications), as well as medium-to-large effects between the two pre-eclampsia groups (effect size >0.75). Assuming a CV of 0.5, these effect sizes correspond to fold changes of approximately 1.61–2.15 for increases in biomarker levels, or 0.47–0.62 for decreases, respectively.

All statistical tests were two-sided, with a significance level set at 5%. Statistical analyses were performed using SAS/STAT® Software, version 9.4 (SAS Institute Inc., Cary, NC, USA) and R software, version 4.4.1 (R Core Team, Vienna, Austria).

### Role of funders

The funding sources had no role in study design, data collection, data analysis, data interpretation, or writing of the report.

## Results

**Fig. 1:**
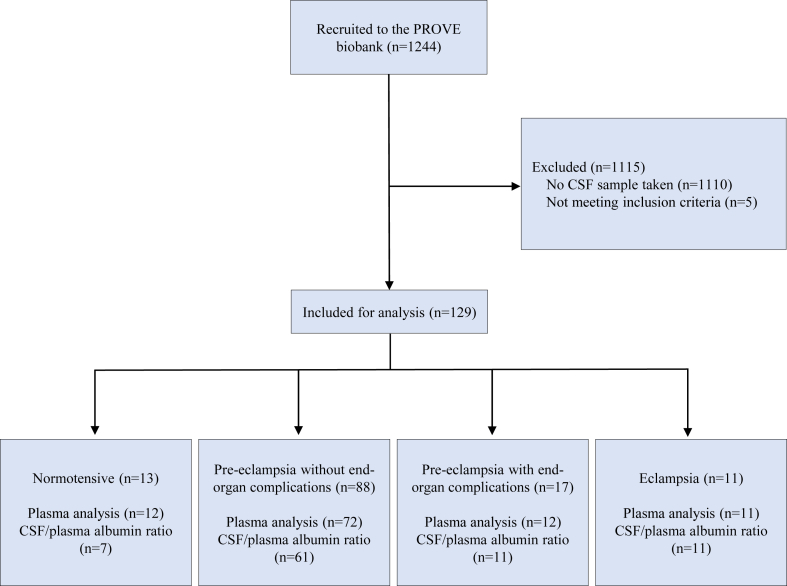
Flow chart of the study cohort illustrating the number of participants with normotensive pregnancies, pre-eclampsia without end-organ complications, pre-eclampsia with end-organ complications, and eclampsia. The number of analyses performed is also presented.

Between March 2021 and June 2023, 1244 women in total were included in the PROVE biobank. Of these, 129 women had cerebrospinal fluid and/or plasma samples collected. Of these, eleven women had eclampsia, 17 had pre-eclampsia with end-organ complications, 88 had pre-eclampsia without complications, and 13 had normotensive pregnancies (Fig. 1). Pre-eclampsia with end-organ complications included seven with pulmonary oedema, seven with HELLP syndrome, and three with severe renal impairment (creatinine >120 μmol/L). Three women with eclampsia also had pulmonary oedema and one had HELLP syndrome. Three had recurrent eclampsia.

### Background characteristics

Women with eclampsia and pre-eclampsia had a lower mean age, were more likely to be nulliparous and needed an emergency caesarean section. Women with pre-eclampsia and end-organ complications, but not eclampsia, gave birth at an earlier gestation and their infants had lower mean birthweights (Table 1). None of the women who experienced eclampsia had chronic hypertension.

**Table 1:** Characteristics of the study cohort at enrolment.

	Normotensive (n = 13)	Pre-eclampsia without end-organ complications (n = 88)	Pre-eclampsia with end-organ complications (n = 17)	Eclampsia (n = 11)
Age (years), mean (SD)	32.5 (6.3)	28.6 (5.9)	28.4 (8.0)	26.3 (6.9)
Body mass index (kg/m^2^), mean (SD)	31.3 (6.3)	29.7 (7.1)	34.9 (8.1)	29.2 (5.0)
Nulliparous, n (%)	2 (15%)	36 (41%)	7 (41%)	6 (55%)
HIV, n (%)	3 (23%)	15 (17%)	1 (6%)	2 (18%)
Smoking during pregnancy, n (%)	0 (0%)	11 (13%)	2 (13%)	1 (9%)
Alcohol use during pregnancy, n (%)	0 (0%)	3 (3%)	2 (12%)	0 (0%)
Chronic hypertension, n (%)	0 (0%)	7 (8%)	2 (12%)	0 (0%)
Pulmonary oedema, n (%)	0	0	7 (41%)	3 (27%)
HELLP syndrome, n (%)	0	0	7 (41%)	1 (9%)
Severe renal impairment (creatinine >120 μmol/L), n (%)	0	0	3 (18%)	0
Placental abruption, n (%)	0	0	1 (6%)	0
Recurrent eclampsia, n (%)	0	0	0	3 (27%)
Elective or non-urgent caesarean section, n (%)	8 (62%)	17 (20%)	6 (38%)	1 (9%)
Emergency caesarean section, n (%)	5 (38%)	70 (80%)	10 (63%)	10 (91%)
Gestation at delivery (weeks + days), median (IQR)	38 + 3 (36 + 2–40 + 4)	36 + 3 (32 + 1–38 + 5)	33 + 4 (30 + 2–38 + 5)	37 + 2 (34 + 1–39 + 4)
Liveborn infant, n (%)	13 (100%)	88 (100%)	17 (100%)	11 (100%)
Birthweight (kg), mean (SD)	2.9 (0.8)	2.2 (0.9)	2.0 (0.8)	2.5 (0.7)

Numeric variables are presented as mean with standard deviation (SD) or median with interquartile range (IQR), while categorical variables are reported as counts and percentages.

Missing data are as follows (by group: normotensive, pre-eclampsia without end-organ complications, pre-eclampsia with end-organ complications, and eclampsia): body mass index – 3/3/1/1; smoking status – 0/0/1/0; chronic hypertension – 0/1/0/0; emergency caesarean section – 0/1/1/0; and birthweight – 0/1/0/0.

Abbreviations: HELLP, haemolysis, elevated liver enzymes, low platelet count; HIV, human immunodeficiency virus; IQR, interquartile range; SD, standard deviation.

### Blood-brain barrier injury

**Fig. 2:**
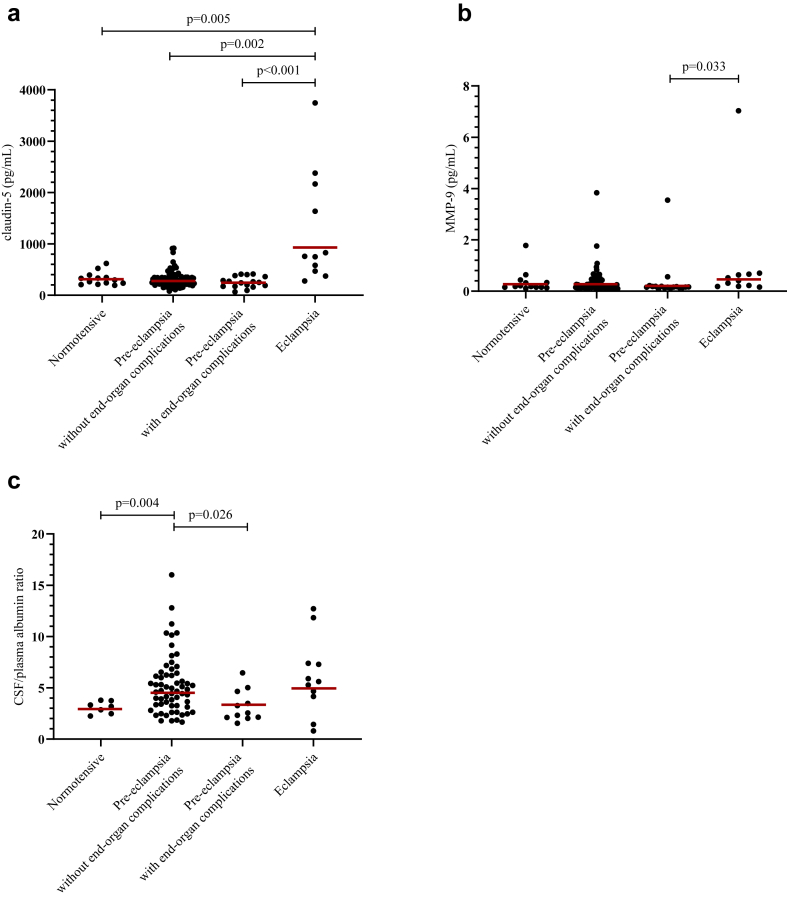
Distribution of blood-brain barrier injury markers in cerebrospinal fluid (CSF) across participant groups. Claudin-5 (**a**), matrix metalloprotease-9 (MMP-9) (**b**), and CSF/plasma albumin ratio (**c**) are shown for normotensive women, women with pre-eclampsia (without and with end-organ complications), and women with eclampsia. Points represent observed values, and red lines indicate geometric means. [p-values were derived using Welch's analysis of covariance on log-transformed data].

Claudin-5 concentrations in cerebrospinal fluid were 4.0-fold higher in women with eclampsia compared to women with pre-eclampsia with end-organ complications (95% CI 2.12–7.47) and a 3.1-fold increased concentration compared to women with pre-eclampsia without end-organ complications (95% CI 1.70–5.61). Women with eclampsia also had a 2.7-fold increase compared to normotensive controls (95% CI 1.43–5.11) (Fig. 2a, Table 2).

**Table 2:** Pairwise comparisons of blood-brain barrier injury markers in cerebrospinal fluid and CSF/plasma ratio among women with normotensive pregnancies, pre-eclampsia without end-organ complications, pre-eclampsia with end-organ complications, and eclampsia.

Biomarker	Reference	Fold change (95% CI) vs reference		
		Pre-eclampsia without end-organ complications (n = 88)	Pre-eclampsia with end-organ complications (n = 17)	Eclampsia (n = 11)
Claudin-5	Normotensive (n = 13)	0.88 (0.64, 1.20) p = 0.37	0.68 (0.46, 1.01) p = 0.054	2.70 (1.43, 5.11) **p = 0.005**
	Pre-eclampsia without end-organ complications	–	0.78 (0.58, 1.04) p = 0.085	3.09 (1.70, 5.61) **p = 0.002**
	Pre-eclampsia with end-organ complications	–	–	3.97 (2.12, 7.47) **p < 0.001**
MMP-9	Normotensive (n = 13)	0.80 (0.42, 1.55) p = 0.48	0.64 (0.29, 1.38) p = 0.24	1.52 (0.64, 3.63) p = 0.32
	Pre-eclampsia without end-organ complications	–	0.79 (0.47, 1.33) p = 0.36	1.89 (0.95, 3.78) p = 0.067
	Pre-eclampsia with end-organ complications	–	–	2.39 (1.08, 5.30) **p = 0.033**
CSF/plasma albumin ratio	Normotensive (n = 7)	1.52 (1.17, 1.98) **p = 0.004**	1.05 (0.73, 1.51) p = 0.78	1.55 (0.82, 2.96) p = 0.16
	Pre-eclampsia without end-organ complications	–	0.69 (0.50, 0.95) **p = 0.026**	1.02 (0.54, 1.92) p = 0.95
	Pre-eclampsia with end-organ complications	–	–	1.48 (0.76, 2.87) p = 0.22

Analyses adjusted for plate differences, maternal age, BMI, HIV, and chronic hypertension. Bold font indicates p-values below 0.05.

Statistical analyses were conducted using Welch's analysis of covariance (ANCOVA) on log-transformed variables to account for unequal variances across groups. Results are expressed as fold changes compared to the reference group, with 95% confidence intervals. Adjustments were made for plate differences, maternal age, BMI, HIV status, and chronic hypertension.

Abbreviations: CI, confidence interval; CSF, cerebrospinal fluid; MMP-9, matrix metalloprotease 9.

Women with pre-eclampsia, with or without end-organ complications, had no significant differences in claudin-5 cerebrospinal fluid concentrations compared to normotensive controls.

MMP-9 was increased 2.4-fold in cerebrospinal fluid when women with eclampsia was compared to women with pre-eclampsia with end-organ complications (95% CI 1.08–5.30) (Fig. 2b, Table 2). There were no other significant differences in MMP-9 concentrations.

There was no difference in cerebrospinal fluid to plasma albumin ratio in women with eclampsia compared to normotensive controls. Women with pre-eclampsia with end-organ complications had a 30% lower albumin ratio compared to those without end-organ complications (fold change 0.69, 95% CI 0.50–0.95). Women with pre-eclampsia without end-organ complications had a 1.5-fold increased albumin ratio compared to normotensive controls (95% CI 1.17–1.98) (Fig. 2c, Table 2).

Descriptive and unadjusted analyses are presented in Supplementary Tables S3 and S4.

### Neuroinflammation

#### Inflammatory recruitment

**Fig. 3:**
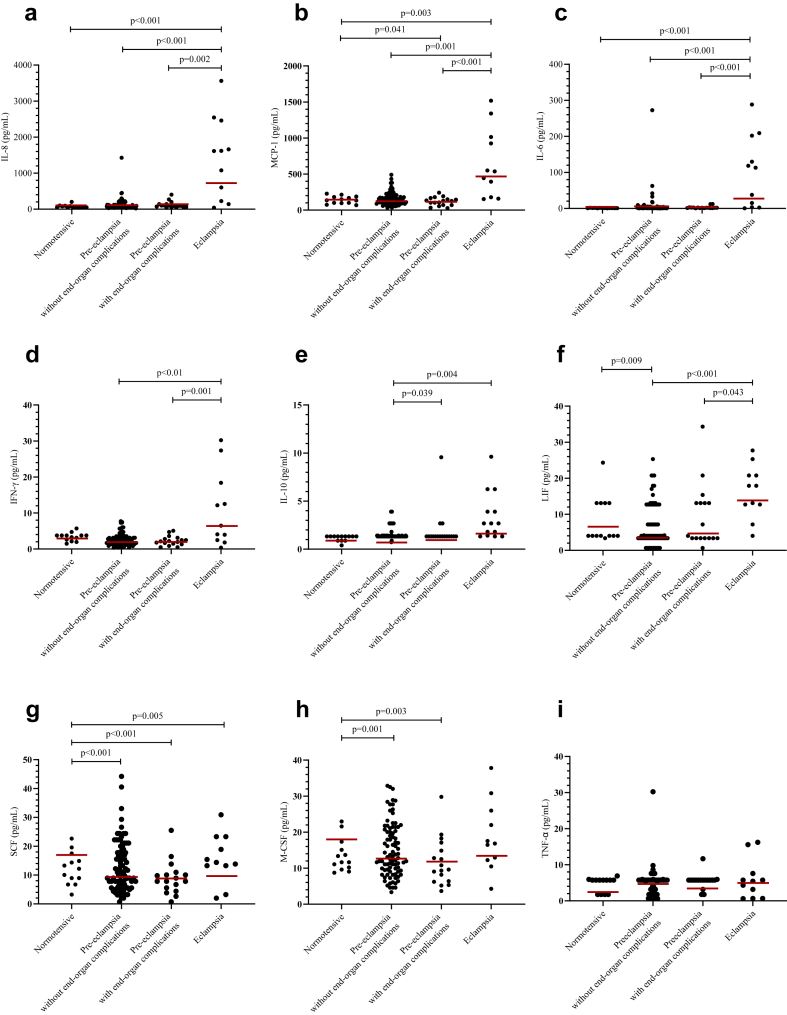
Distribution of inflammatory cytokines interleukin and growth factors in cerebrospinal fluid across participant groups. Shown are interleukin-8 (IL-8) (**a**), monocyte chemoattractant protein 1 (MCP-1) (**b**), interleukin-6 (IL-6) (**c**), interferon-γ (IFN-γ) (**d**), interleukin-10 (IL-10) (**e**), leukaemia inhibitory factor (LIF) (**f**), stem cell factor (SCF) (**g**), macrophage colony stimulating factor (M-CSF) (**h**), and tumour necrosis factor α (TNF-α) (**i**). Data are presented for normotensive women, women with pre-eclampsia (without and with end-organ complications), and women with eclampsia. Points represent observed values, and red lines indicate geometric means. [p-values were derived using log-normal Tobit regression for IL-6, TNF-α, IFN-γ, LIF, SCF, and IL-10, and Welch's analysis of covariance for IL-8, MCP-1, and M-CSF, all on log-transformed data].

Eclampsia was associated with increased cerebrospinal fluid concentrations of several chemokines reflecting inflammatory recruitment. Women with eclampsia had a 6.0-fold increase in IL-8 concentrations when compared to women with pre-eclampsia with end-organ complications (95% CI 2.29–15.55), a 7.0-fold increase when compared to women with pre-eclampsia without end-organ complications (95% CI 2.80–17.75), and a 7.2-fold increase when compared to normotensive controls (95% CI 2.76–18.75) (Fig. 3a, Table 3). Women with eclampsia also had a 4.3-fold increase in monocyte chemoattractant protein 1 (MCP-1) when compared to women with pre-eclampsia with end-organ complications (95% CI 2.30–8.19), a 3.3-fold increase when compared to women with pre-eclampsia without end-organ complications (95% CI 1.82–5.92), and a 2.8-fold increase (95% CI 1.50–5.36) compared to normotensive controls (Fig. 3b, Table 3). Women with pre-eclampsia with end-organ complications had 35% lower levels of MCP-1 compared to normotensive controls (95% CI 0.44–0.98). Growth-regulated protein alpha (GRO-α) was detected in 5/11 women with eclampsia, none with pre-eclampsia with end-organ complications, 1/88 (1.2%) with pre-eclampsia without end-organ complications, and none of the normotensive controls (Supplementary Table S5).

**Table 3:** Pairwise comparisons of inflammatory cytokines in cerebrospinal fluid among women with normotensive pregnancies, pre-eclampsia without end-organ complications, pre-eclampsia with end-organ complications, and eclampsia.

Biomarker	Reference	Fold change (95% CI) vs reference		
		Pre-eclampsia without end-organ complications (n = 88)	Pre-eclampsia with end-organ complications (n = 17)	Eclampsia (n = 11)
IL-8	Normotensive (n = 13)	1.02 (0.71, 1.47) p = 0.91	1.21 (0.76, 1.91) p = 0.41	7.19 (2.76, 18.75) **p < 0.001**
	Pre-eclampsia without end-organ complications	–	1.18 (0.82, 1.70) p = 0.35	7.04 (2.80, 17.75) **p < 0.001**
	Pre-eclampsia with end-organ complications	–	–	5.96 (2.29, 15.55) **p = 0.002**
MCP-1	Normotensive	0.86 (0.63, 1.19) p = 0.35	0.65 (0.44, 0.98) **p = 0.041**	2.84 (1.50, 5.36) **p = 0.003**
	Pre-eclampsia without end-organ complications	–	0.76 (0.55, 1.05) p = 0.089	3.29 (1.82, 5.92) **p = 0.001**
	Pre-eclampsia with end-organ complications	–	–	4.34 (2.30, 8.19) **p < 0.001**
IL-6	Normotensive	1.46 (0.61, 3.50) p = 0.39	1.71 (0.61, 4.81) p = 0.31	20.7 (6.51, 65.7) **p < 0.001**
	Pre-eclampsia without end-organ complications	–	1.17 (0.58, 2.38) p = 0.66	14.14 (6.25, 31.95) **p < 0.001**
	Pre-eclampsia with end-organ complications	–	–	12.06 (4.33, 33.62) **p < 0.001**
IFN-γ	Normotensive	0.71 (0.41, 1.22) p = 0.21	0.68 (0.36, 1.28) p = 0.23	1.95 (0.95, 3.99) p = 0.068
	Pre-eclampsia without end-organ complications	–	0.96 (0.62, 1.48) p = 0.84	2.75 (1.66, 4.56) **p < 0.001**
	Pre-eclampsia with end-organ complications	–	–	2.87 (1.52, 5.40) **p = 0.001**
IL-10	Normotensive	0.74 (0.36, 1.50) p = 0.40	1.29 (0.61, 2.71) p = 0.51	1.45 (0.66, 3.20) p = 0.35
	Pre-eclampsia without end-organ complications	–	1.75 (1.03, 2.97) **p = 0.039**	1.97 (1.24, 3.13) **p = 0.004**
	Pre-eclampsia with end-organ complications	–	–	1.13 (0.60, 2.14) p = 0.71
LIF	Normotensive	0.35 (0.16, 0.77) **p = 0.009**	0.49 (0.19, 1.28) p = 0.15	1.29 (0.46, 3.63) p = 0.63
	Pre-eclampsia without end-organ complications	–	1.43 (0.73, 2.78) p = 0.29	3.74 (1.81, 7.74) **p < 0.001**
	Pre-eclampsia with end-organ complications	–	–	2.62 (1.03, 6.66) **p = 0.043**
SCF	Normotensive	0.48 (0.34, 0.70) **p < 0.001**	0.43 (0.28, 0.67) **p < 0.001**	0.50 (0.31, 0.81) **p = 0.005**
	Pre-eclampsia without end-organ complications	–	0.90 (0.67, 1.20) p = 0.47	1.03 (0.73, 1.46) p = 0.85
	Pre-eclampsia with end-organ complications	–	–	1.15 (0.75, 1.77) p = 0.51
M-CSF	Normotensive	0.67 (0.53, 0.83) **p = 0.001**	0.61 (0.45, 0.83) **p = 0.003**	0.73 (0.52, 1.03) p = 0.069
	Pre-eclampsia without end-organ complications	–	0.92 (0.72, 1.19) p = 0.51	1.10 (0.83, 1.46) p = 0.48
	Pre-eclampsia with end-organ complications	–	–	1.19 (0.84, 1.69) p = 0.31
TNF-α	Normotensive	0.83 (0.38, 1.81) p = 0.64	0.93 (0.36, 2.40) p = 0.88	1.06 (0.40, 2.82) p = 0.91
	Pre-eclampsia without end-organ complications	–	1.12 (0.57, 2.20) p = 0.75	1.27 (0.67, 2.43) p = 0.46
	Pre-eclampsia with end-organ complications	–	–	1.14 (0.47, 2.79) p = 0.77

Analyses adjusted for plate differences, maternal age, BMI, HIV status, and chronic hypertension. Bold font indicates p-values below 0.05.

Statistical analyses for IL-6, TNF-α, IFN-γ, LIF, SCF, and IL-10 in cerebrospinal fluid were conducted using log-normal Tobit regression to account for censoring of values below the detection limit. Analyses for IL-8, MCP-1, and M-CSF were performed using Welch's analysis of covariance on log-transformed variables to address unequal variances across groups. Results are presented as fold changes relative to the reference group, with 95% confidence intervals, adjusted for plate differences, maternal age, BMI, HIV status, and chronic hypertension.

Abbreviations: CI, confidence interval; IFN, interferon; IL, interleukin; LIF, leukaemia inhibitory factor; M-CSF, macrophage colony-stimulating factor; MCP-1, monocyte chemoattractant protein-1; SCF, stem cell factor; TNF, tumour necrosis factor.

#### Cytotoxic signals

Several cytotoxic signalling cytokines were elevated in women with eclampsia. Cerebrospinal fluid concentrations of IL-6 were 12-times higher when compared to pre-eclampsia with end-organ complications (95% CI 4.33–33.62), 14-times higher when compared to pre-eclampsia without end-organ complications (95% CI 6.25–31.95), and 21-times higher compared to controls (95% CI 6.51–65.70) (Fig. 3c, Table 3). Women with eclampsia had 2.9-fold higher IFN-γ concentrations when compared to women with pre-eclampsia with end-organ complications (95% CI 1.52–5.40) and 2.8-fold higher concentrations when compared to women with pre-eclampsia without end-organ complications (95% CI 1.66–4.56) (Fig. 3d, Table 3).

β-nerve growth factor (β-NGF), a sign of neuronal injury, was found in 6/11 women with eclampsia, 1/17 women with pre-eclampsia with end-organ complications, 32/88 women with pre-eclampsia without end-organ complications, and none of the normotensive controls (Supplementary Table S5).

#### Immune modulation

In acute inflammation, inflammatory modulating signals counteract the inflammatory response. Women with eclampsia had a 1.8-fold increase in IL-10 concentrations when compared to women with pre-eclampsia with end-organ complications (95% CI 1.03–2.97) and a 2-fold increase when compared to women with pre-eclampsia without end-organ complications (95% CI 1.24–3.13) (Fig. 3e, Table 3). The neuropoietic cytokine leukaemia inhibitory factor (LIF) regulating pro- and anti-inflammatory actions was 2.6-times higher in women with eclampsia compared to women with pre-eclampsia with end-organ complications (95% CI 1.03–6.66) and 3.8-times higher in women with eclampsia when compared to women with pre-eclampsia without end-organ complications (95% CI 1.81–7.74) (Fig. 3f, Table 3). Women with pre-eclampsia with end-organ complications had similar concentrations of LIF compared to normotensive controls. LIF was reduced by 65% in women with pre-eclampsia without end-organ complications compared to normotensive controls (fold change 0.35, 95% CI 0.16–0.77).

Neuroprotective cytokine stem cell factor (SCF) concentrations were 50% lower in women with eclampsia (fold change 0.50, 95% CI 0.31–0.81), 57% lower in women with pre-eclampsia with end-organ complications (fold change 0.43, 95% CI 0.28–0.67), and 52% lower in women with pre-eclampsia without end-organ complications (fold change 0.48, 95% CI 0.31–0.81) compared to normotensive controls (Fig. 3g, Table 3). Macrophage colony stimulating factor (M-CSF) concentrations were reduced by 39% in women with pre-eclampsia with end-organ complications (fold change 0.61, 95% CI 0.45–0.83%) and by 33% in women with pre-eclampsia without end-organ complications (fold change 0.67, 95% CI 0.53–0.83) when compared to normotensive controls (Fig. 3h, Table 3).

TNF-α concentrations were similar across groups (Fig. 3i, Table 3).

A complete list of results from the multiplex analyses are presented in Supplementary Tables S3 and S4.

### Correlation analysis

We found weak positive correlations between all blood-brain barrier injury markers in cerebrospinal fluid (Table 4, Supplementary Figure S1). There was a high positive correlation between the pro-inflammatory cytokines IL-6 and IL-8 (r = 0.74, 95% CI 0.65–0.81) and a weak positive correlation between TNF-α and IL-6 (r = 0.22, 95% CI 0.02–0.40) (Table 5, Supplementary Figure S2). There was no correlation between inflammatory markers in cerebrospinal fluid and plasma (Supplementary Table S6, Supplementary Figure S3).

**Table 4:** Correlations between cerebrospinal fluid (CSF) concentrations of claudin-5, MMP-9, and CSF/plasma albumin ratio.

	Claudin-5	MMP-9	Albumin CSF/plasma ratio
Claudin-5	–	0.18 (0.01, 0.34) **p = 0.039**	0.23 (0.02, 0.42) **p = 0.030**
MMP-9	0.18 (0.01, 0.34) **p = 0.039**	–	0.26 (0.06, 0.45) **p = 0.012**
CSF/plasma albumin ratio	0.23 (0.02, 0.42) **p = 0.030**	0.26 (0.06, 0.45) **p = 0.012**	–

Values are presented as Pearson correlation coefficients with 95% confidence intervals, calculated from log-transformed data. Bold font indicates p-values below 0.05.

Abbreviations: CSF, cerebrospinal fluid; MMP-9, matrix-metalloprotease 9.

**Table 5:** Correlations between inflammatory cytokines IL-6, IL-8, and TNF-α in cerebrospinal fluid.

	IL-6	IL-8	TNF-α
IL-6	–	0.74 (0.65, 0.81) **p < 0.001**	0.22 (0.02, 0.40) **p = 0.031**
IL-8	0.74 (0.65, 0.81) **p < 0.001**	–	0.18 (−0.01, 0.35) p = 0.061
TNF-α	0.22 (0.02, 0.40) **p = 0.031**	0.18 (−0.01, 0.35) p = 0.061	–

Values are presented as bivariate correlation coefficients with 95% confidence intervals, calculated using log-Gaussian profile likelihood to account for the censoring of values below the lower detection limit. Bold font indicates p-values below 0.05.

Abbreviations: IL, interleukin; TNF, tumour necrosis factor.

## Discussion

Eclampsia is associated with injury of the blood-brain barrier and increased neuroinflammatory activity, independent of systemic inflammation. Increased signals related to inflammatory recruitment, cytotoxic pathways, inflammatory modulation, and reduced neuroprotection all indicate an acute cerebral insult in eclampsia, potentially leading to neuronal cell death. Women with pre-eclampsia showed little to no blood-brain barrier injury and only modest neuroinflammatory changes.

In our prior small pilot study, eclampsia was associated with increased concentrations IL-6, IL-8, and TNF-α in cerebrospinal fluid and an elevated albumin ratio, suggesting blood-brain barrier injury and inflammation.^20^ Our findings provide evidence that eclampsia is associated with blood-brain barrier injury and increased neuroinflammation.

Blood-brain barrier injury in pre-eclampsia is not well studied. Two studies of pre-eclampsia, without cases of eclampsia, reported no significant differences whereas we found differences in our studies.^19^^,^^20^^,^^28^ This variance is likely due to differences in disease severity.

We showed increased concentrations of claudin-5 in cerebrospinal fluid in eclampsia, indicating cerebrovascular tight-junction disruption as a potential cause of blood-brain barrier injury. A preclinical study suggests that small extracellular vesicles from pre-eclampsia may lower claudin-5 levels on blood-brain barrier endothelial cells.^18^ Increased cerebrospinal fluid concentrations and/or dislocated claudin-5 have been reported after intracranial haemorrhage and epilepsy.^29^, ^30^, ^31^ Claudin-5 may be removed from endothelial cell-surfaces by proteolytic cleavage executed by matrix metalloproteases. Matrix metalloproteases were increased in the cerebrospinal fluid in eclampsia. MMP-9 is well studied in blood-brain barrier injury, particularly for neurodegenerative disorders.^32^ MMP-9 can be activated by microglia, suggesting crosstalk between neuroinflammatory signals and blood-brain barrier endothelial cells.^33^ Claudin-5 disruption may occur through the actions of the chemokine MCP-1, which is elevated in eclampsia. MCP-1 is released from the blood-brain barrier endothelial cells and binds to the CCR2 receptor on microglia, neurons, astrocytes, and endothelial cells.^34^ The binding of MCP-1 to CCR2 triggers alterations in the cytoskeleton and reduces the expression of tight junction proteins on the cell surface, potentially worsening blood-brain barrier injury and serving as an underlying cause of blood-brain barrier injury.^35^ Increased MCP-1 signalling have also been associated with other neurodegenerative disorders.^36^

IL-8 and GRO-α were elevated in the cerebrospinal fluid of women with eclampsia. Both these chemokines are elevated in other neurodegenerative diseases where they act as recruitment signals for inflammatory mediators.^37^^,^^38^ Chemokines are secreted by endothelial cells and immunoreactive glial cells to enhance inflammatory pathways leading to cytotoxicity.^35^^,^^39^ IL-8 induces migration and activation of microglia, which subsequently release cytotoxic IL-6, amplifying neuroinflammatory pathways.^40^

The proinflammatory cytokines IL-6 and IFN-γ originate from activated microglia.^38^^,^^41^^,^^42^ They initiate and drive the acute inflammatory responses, leading to neurotoxicity in neurodegenerative disorders.^43^ These cytokines were increased in eclampsia. Further evidence of neuronal injury in eclampsia is demonstrated by higher concentrations of β-NGF indicating dysregulated metabolism.^44^ β-NGF is associated with neuroinflammation in Alzheimer's disease and other neurodegenerative disorders, leading to cognitive decline.^44^^,^^45^

After acute inflammation and cell injury immune modulation occurs. Acutely activated microglia express anti-inflammatory and neuroprotective cytokines such as IL-10 and LIF. IL-10 and LIF were increased in women with eclampsia. A similar acute anti-inflammatory response pattern has been reported in patients with Alzheimer's disease, cerebral ischaemia, and traumatic brain injury.^46^, ^47^, ^48^, ^49^ The reduced concentrations of SCF and M-CSF in women with eclampsia suggest impaired neuroregeneration, which may contribute to long-term neurological sequelae.^50^^,^^51^

The blood-brain barrier injury, the increased inflammatory response, and the cytotoxic signalling in the brain in eclampsia are concerning. This is particularly relevant as women with pre-eclampsia and eclampsia have been shown to have silent infarcts, impaired cognition, and subsequent risk of stroke, epilepsy, and dementia.^6^, ^7^, ^8^^,^^52^, ^53^, ^54^, ^55^, ^56^, ^57^, ^58^, ^59^ In a recent study, we found that 80% of women with eclampsia show signs of vasogenic cerebral oedema, compared to 20% of women with preeclampsia.^59^ Given the acute neuroinflammatory phenotype in women with eclampsia characterised in this current study, cerebral oedema is possibly influenced by neuroinflammation and/or trigger further neuroinflammation in eclampsia. This hypothesis warrants further investigation.

Our findings demonstrate parallels between the blood-brain barrier injury and neuroinflammation observed in eclampsia and those observed in neurodegenerative diseases and traumatic brain injury. Clinical surveillance and long-term follow-up are advised for these neurological conditions in several clinical guidelines.^60^^,^^61^ Surveillance and long-term follow-up of women with eclampsia are not widely recommended in national or international guidelines. Follow-up of women who experienced eclampsia should be considered to identify any long-term sequelae and provide appropriate interventions.

The lack of correlation between cytokines in cerebrospinal fluid and plasma show that the neuroinflammatory state in pre-eclampsia and eclampsia likely results from inflammatory factors being produced within the brain. Systemic inflammation seems to play a lesser role in neuroinflammation.

It is unclear what initiates the blood-brain barrier injury in pre-eclampsia and eclampsia. Potential contributors include defective cerebral autoregulation, leading to hypoxia and injury to the blood-brain barrier,^62^ and unknown signalling proteins in the circulation such as angiogenic factors that may play an important role in blood-brain barrier activation. Future studies should focus on identifying the initiators of blood-brain barrier injury to discover potential drug targets for neuroprotection. Researchers should examine the effects of neuroinflammation through comprehensive analyses of neuronal injury in both the short- and long-term. This includes both human studies and pre-eclampsia- and eclampsia-like *in vivo* and *in vitro* models.^11^^,^^63^

This study investigates rare biological samples: paired cerebrospinal fluid and plasma samples from women with eclampsia. To assess potential selection bias, we compared women included in the current study to larger cohorts recruited to the PROVE biobank, as reported in previous publications from our group.^21^^,^^59^^,^^62^ Overall, maternal baseline characteristics and neonatal outcomes were similar between our study cohort and the broader PROVE population. The prevalence and pattern of maternal complications among women with eclampsia, including the proportion of recurrent eclampsia cases, were also consistent across studies (Supplementary Table S7).^21^^,^^59^ We successfully included a well characterised cohort and applied extensive and sophisticated analyses such as multiplex cytokine assays, to gain a deeper understanding of blood-brain barrier injury and neuroinflammation in eclampsia. These results provide thorough insight into the pathophysiology behind cerebral complications in pre-eclampsia.

Our study has limitations. Samples were only drawn after the onset of eclampsia. We lack information on blood-brain barrier injury or neuroinflammation before the onset of seizures. Thus, we cannot determine whether the observed changes are causes or consequences of eclamptic seizures. Despite being a unique study with a substantial number of women with eclampsia, the low absolute numbers may still pose a limitation and introduce type 2 errors, i.e., false negative findings. Despite this limitation, we saw a clear pattern of inflammatory and blood-brain barrier-proteins altered in eclampsia.

Women with eclampsia experience acute neuroinflammation and blood-brain barrier injury, comparable to patterns observed in neurodegenerative diseases such as acute traumatic brain injury, stroke or Alzheimer's disease. Clinical follow-up of women with eclampsia postpartum should be considered. The source of neuroinflammation lies within cerebral tissue, with both neuroinflammation and blood-brain barrier injury most likely progressing simultaneously, triggered by endothelial dysfunction and altered inflammatory signalling across the neurovascular unit. Women with pre-eclampsia had subtle signals of blood-brain barrier injury and neuroinflammation, potentially indicating a pre-clinical state on the progression to eclampsia.

## Contributors

VB, OTH, JE, SPW, ST, CC, and LB conceptualised the study. JE, CC, and LB provided overall supervision. VB, JE, and LB obtained funding for the study. VB, OTH, JE, CC, and LB had direct access to the full dataset and take responsibility for the integrity of the data and the accuracy of the data analysis. VB, OTH, JE, and HI acquired the data and performed the statistical analysis. SS, JV, JB, HZ, and KB provided administrative, technical or material support. VB, OTH, JE, and LB verified the underlying data. VB wrote the first draft of the report with input from LB and CC. All authors edited, validated, critically revised the manuscript, and approved the final version of the manuscript. All authors had full access to all the data in the study and accept responsibility to submit for publication.

## Data sharing statement

Anonymised data will be shared on request from a qualified researcher after ethical approval and according to patient informed consent with investigators support.

## Declaration of interests

HZ has served at scientific advisory boards and/or as a consultant for Abbvie, Acumen, Alector, Alzinova, ALZpath, Amylyx, Annexon, Apellis, Artery Therapeutics, AZTherapies, Cognito Therapeutics, CogRx, Denali, Eisai, Enigma, LabCorp, Merry Life, Nervgen, Novo Nordisk, Optoceutics, Passage Bio, Pinteon Therapeutics, Prothena, Quanterix, Red Abbey Labs, reMYND, Roche, Samumed, Siemens Healthineers, Triplet Therapeutics, and Wave, has given lectures sponsored by Alzecure, BioArctic, Biogen, Cellectricon, Fujirebio, Lilly, Novo Nordisk, Roche, and WebMD, and is together with KB a co-founder of Brain Biomarker Solutions in Gothenburg AB (BBS), which is a part of the GU Ventures Incubator Program (outside submitted work). HZ is chair of the Alzheimer's Association Global Biomarker Standardisation Consortium and chair of the IFCC WG-BND. HZ is a Wallenberg Scholar and a Distinguished Professor at the Swedish Research Council supported by grants from the Swedish Research Council (#2023-00356; #2022-01018; and #2019-02397), the European Union's Horizon Europe research and innovation programme under grant agreement No 101053962, Swedish State Support for Clinical Research (#ALFGBG-71320), the Alzheimer Drug Discovery Foundation (ADDF), USA (#201809-2016862), the AD Strategic Fund and the Alzheimer's Association (#ADSF-21-831376-C, #ADSF-21-831381-C, #ADSF-21-831377-C, and #ADSF-24-128438-C), the Bluefield Project, Cure Alzheimer's Fund, the Olav Thon Foundation, the Erling-Persson Family Foundation, Stiftelsen fӧr Gamla Tjӓnarinnor, Hjӓrnfonden, Sweden (#FO2022-0270), the European Union's Horizon 2020 research and innovation programme under the Marie Skłodowska-Curie grant agreement No 860197 (MIRIADE), the European Union Joint Programme – Neurodegenerative Disease Research (JPND2021-00694), the National Institute for Health and Care Research University College London Hospitals Biomedical Research Centre, the UK Dementia Research Institute at UCL (UKDRI-1003), and an anonymous donor.

KB has served at scientific advisory boards and/or as a consultant for Abbvie, AC Immune, ALZpath, Aribio, Beckman Coulter, BioArctic, Biogen, Eisai, Lilly, Neurimmune, Ono Pharma, Prothena, Roche Diagnostics, Sanofi, Siemens Healthineers, Novartis, and Julius Clinical. KB has received payments from Biogen, Eisai, and Roche Diagnostics for participation in educational programs. KB has received grants paid to the institute from Swedish Research Council (#2017-00915 and #2022-00732), the Swedish state under the agreement between the Swedish government and the County Councils, the ALF-agreement (#ALFGBG-965240 and #ALFGBG-1006418), the Swedish Alzheimer Foundation (#AF-930351, #AF-939721, #AF-968270, and #AF-994551), Hjärnfonden, Sweden (#ALZ2022-0006, #FO2024-0048-TK-130, and FO2024-0048-HK-24), the Alzheimer's Association 2021 Zenith Award (ZEN-21-848495), the Alzheimer's Association 2022-2025 Special Grant (SG-23-1038904 QC), La Fondation Recherche Alzheimer (FRA), Paris, France, the Kirsten and Freddy Johansen Foundation, Copenhagen, Denmark, Familjen Rӧnstrӧms Stiftelse, Stockholm, Sweden To the Institute.

LB has collaborated with Roche, PerkinElmer, and Thermo Fischer as sponsors for PlGF and sFlt-1 reagents (IMPACT study). LB has obtained reimbursement for lecture by iLab Medical and Thermo Fisher, expert opinion from Homburg and Partner. LB has received trial drug and placebo from Merck.

## Appendix ASupplementary data

Supplementary Materials Supplementary Figure S1: Correlations between blood-brain barrier injury markers. Supplementary Figure S2: Correlations between inflammatory cytokines in CSF. Supplementary Figure S3: Correlations between inflammatory cytokines in CSF and plasma.
