# Supplementary material for: Blood-brain barrier injury and neuroinflammation in pre-eclampsia and eclampsia
Source: eBioMedicine. 2025 May 8;116:105742. doi: 10.1016/j.ebiom.2025.105742 (PMC12136835; doi:10.1016/j.ebiom.2025.105742)
Supplement: Supplementary Materials — Supplementary Figure S1: Correlations between blood-brain barrier injury markers. Supplementary Figure S2: Correlations between inflammatory cytokines in CSF. Supplementary Figure S3: Correlations between inflammatory cytokines in CSF and plasma. [file mmc1.docx]

**Supplementary material for “Blood Brain Barrier Injury and Neuroinflammation in Pre-eclampsia and Eclampsia”**

**Table of Contents**

[**Supplementary table 1.** Definition of study groups. 2](#_Toc194950674)

[**Supplementary table 2.** List of analysed inflammatory cytokines, chemokines, and growth factors. 3](#_Toc194950675)

[**Supplementary table 3.** Descriptive data of blood-brain barrier disruption markers and cytokines in women with normotensive pregnancies, pre-eclampsia without complications, pre-eclampsia with end-organ complications, and eclampsia. 4](#_Toc194950676)

[**Supplementary table 4.** Pairwise comparisons of blood-brain barrier disruption markers and inflammatory cytokines in women with normotensive pregnancies, pre-eclampsia without complications, pre-eclampsia with end-organ complications, and eclampsia, adjusted for plate differences. 8](#_Toc194950677)

[**Supplementary table 5.** Descriptive data of inflammatory cytokines, chemokines and growth factors in CSF and plasma in women with normotensive pregnancies, pre-eclampsia without complications, pre-eclampsia with end-organ complications, and eclampsia, highlighting markers that were not detectable across all study groups. 11](#_Toc194950678)

[**Supplementary table 6.** Correlations between inflammatory cytokines in CSF and plasma. 12](#_Toc194950679)

[**Supplementary table 7.** Comparison of women with eclampsia included in the current study to women with eclampsia recruited to the PROVE biobank as reported in previous publications.^22,60,63^ 13](#_Toc194950680)

[**Supplementary figure 1.** 14](#_Toc194950681)

[**Supplementary figure 2.** 15](#_Toc194950682)

[**Supplementary figure 3.** 16](#_Toc194950683)

[**Supplementary checklist 1:** STROBE Statement—Checklist of items that should be included in reports of ***cross-sectional studies***. 17](#_Toc194950684)

**Supplementary table 1.** Definition of study groups.

| **Group** | **Definition** |
| --- | --- |
| Eclampsia | Generalized tonic-clonic seizures in a woman with pre-eclampsia. |
| Pre-eclampsia with end-organ complications | Systolic blood pressure (SBP) ≥140 mm Hg and/or diastolic blood pressure (DPB) ≥90 mm Hg together with excessive proteinuria (≥2+ on a urinary dipstick and/or albumin / creatinine ratio >8 mmol/mol and/or protein/creatinine ratio >30 mmol/mol in 24-hour urine collection) |
| Pre-eclampsia without end-organ complications | SBP ≥140 mm Hg and/or DPB ≥90 mm Hg together with excessive proteinuria (≥2+ or above on a urinary dipstick and/or albumin / creatinine ratio >8 mmol/mol and/or protein/creatinine ratio >30 mmol/mol in 24-hour urine collection)  + with one or more: maternal death, stroke, cortical blindness, pulmonary edema, acute kidney injury (creatinine > 120 µmol/L), hemolysis, elevated liver enzymes, low platelet count (HELLP) syndrome, liver capsule hematoma or rupture, placental abruption, admission to intensive care unit, intubation and mechanical ventilation (not for childbirth) |
| Normotensive | Blood pressure <140/90 mmHg. |

**Supplementary table 2.** List of analysed inflammatory cytokines, chemokines, and growth factors.

| **Category** | **Analysed molecules** |
| --- | --- |
| Proinflammatory (differentiation/activation) | IL-1β, IL-12 (p40), IL-4, IL-6, IL-12 (p70), IL-17A, Tumour necrosis factor alpha (TNF-α), macrophage inhibitory factor (MIF), TNF-related apoptosis inducing ligand (TRAIL), IL-18, TNF-β, IL-2 receptor alpha (IL2R α) |
| Proinflammatory (proliferation) | Interferon gamma (IFNγ), IL-1α, IL-2, IL-3, IL-5, IL-7, IL-9, Interferon alpha (IFNα), Leukaemia inhibitory factor (LIF), IL-15, IL-17, macrophage colony-stimulating factor (M-CSF), granulocytes-macrophage colony-stimulating factor (GM-CSF), granulocyte colony-stimulating factor (G-CSF) |
| Proinflammatory immune attractant | IL-16, IL-8 (CXCL8), GRO-α (melanoma growth stimulating activity alpha CXCL1), Monocyte chemoattractant protein 3 (MCP-3,CCL7), Interferon induced protein 10 (IP 10, CXCL10), Monocyte chemoattractant protein (MCP-1,CCL2), Monokine induced by IFNγ (MIG, CXCL9), Eotaxin, stromal-cell derived factor 1 (SDF-1α, CXCL 12), Macrophage inflammatory protein alpha (MIP1α, CCL3), Macrophage inflammatory protein 1 beta (MIP1β, CCL4), regulated on activated normal T cells expressed and secreted (RANTES, CCL5), skin-associated chemokine, attracts skin homing T memory cells (CTACK) |
| Anti-inflammatory | IL-13, IL-1Rα, IL-10 |
| Growth factors | Vascular endothelial growth factor (VEGF), fibroblast growth factor 2 (FGF basic), platelet derived growth factor subunit B homodimer (PDGF-BB), beta nerve-growth factor (β-NGF), stem cell factor (SCF), serum stem cell growth factor beta (SCGF-β), hepatocyte growth factor (HGF) |

**Supplementary table 3.** Descriptive data of blood-brain barrier disruption markers and cytokines in women with normotensive pregnancies, pre-eclampsia without complications, pre-eclampsia with end-organ complications, and eclampsia.

|  | **Normotensive (n=13)** | **Pre-eclampsia without end-organ complications (n=88)** | **Pre-eclampsia with end-organ complications (n=17)** | **Eclampsia (n=11)** |
| --- | --- | --- | --- | --- |
| Claudin-5 (pg/mL) |  |  |  |  |
| Geometric mean (CV) | 304.6 (0.42) | 278.6 (0.45) | 227.8 (0.53) | 928.9 (0.98) |
| Median (IQR) | 297.8 (235.2–347.0) | 283.7 (216.3–350.4) | 250.5 (173.0–362.8) | 758.8 (470.0–2167.9) |
| Range | 193.8–618.8 | 81.4–915.3 | 65.0–414.8 | 278.1–3743.5 |
| Missing | 0 | 0 | 0 | 0 |
| MMP-9 (pg/mL) |  |  |  |  |
| Geometric mean (CV) | 0.26 (0.92) | 0.25 (0.95) | 0.19 (1.02) | 0.46 (1.38) |
| Median (IQR) | 0.19 (0.15–0.34) | 0.19 (0.15–0.44) | 0.16 (0.14–0.19) | 0.41 (0.19–0.66) |
| Range | 0.11–1.78 | 0.09–7.94 | 0.09–3.55 | 0.16–7.03 |
| Missing | 0 | 1 | 0 | 0 |
| Albumin CSF/plasma ratio (10^-3^) |  |  |  |  |
| Geometric mean (CV) | 3.04 (0.20) | 4.61 (0.55) | 2.94 (0.47) | 4.80 (1.00) |
| Median (IQR) | 3.2 (2.5–3.7) | 4.7 (3.3–6.3) | 2.5 (2.1–4.6) | 5.6 (4.2–7.4) |
| Range | 2.3–3.8 | 1.7–16.0 | 1.5–6.5 | 0.8–12.7 |
| Missing | 6 | 27 | 6 | 0 |
| IL-8 (pg/mL) |  |  |  |  |
| Geometric mean (CV) | 81.2 (0.46) | 91.1 (0.70) | 106.4 (0.69) | 711.7 (2.19) |
| Median (IQR) | 59.3 (49.2–85.1) | 81.5 (63.7–131.2) | 92.6 (58.3–139.3) | 1613.0 (224.6–2459.5) |
| Range | 39.9–201.1 | 21.1–1426.5 | 35.9–401.3 | 39.9–3561.8 |
| Extrapolated outside range | 0 | 0 | 0 | 0 |
| OOR | 0 | 0 | 0 | 0 |
| Missing | 0 | 0 | 0 | 0 |
| MCP-1 (pg/mL) |  |  |  |  |
| Geometric mean (CV) | 147.0 (0.41) | 132.5 (0.56) | 108.5 (0.62) | 464.8 (0.95) |
| Median (IQR) | 136.0 (100.6–181.6) | 136.6 (95.0–184.5) | 126.1 (70.7–149.3) | 537.5 (178.1–1013.5) |
| Range | 68.0–227.9 | 33.6–490.1 | 27.3–242.1 | 153.4–1518.4 |
| Extrapolated outside range | 0 | 0 | 0 | 0 |
| OOR | 0 | 0 | 0 | 0 |
| Missing | 0 | 0 | 0 | 0 |
| IL-6 (pg/mL) |  |  |  |  |
| Geometric mean (CV) | 1.23 (0.49) | 1.74 (1.87) | 2.09 (0.99) | 27.3 (5.05) |
| Median (IQR) | 1.0 (0.6–1.2) | 1.6 (0.9–3.3) | 1.8 (1.1–2.5) | 113.0 (3.3–202.0) |
| Range | 0.4–2.0 | 0.1–272.7 | <LOD–12.36 | 0.4–288.4 |
| Extrapolated outside range | 2 | 7 | 0 | 1 |
| OOR | 0 | 3 | 1 | 0 |
| Missing | 0 | 0 | 0 | 0 |
| IFN-γ (pg/mL) |  |  |  |  |
| Geometric mean (CV) | 2.98 (0.37) | 1.98 (0.85) | 1.79 (0.73) | 6.28 (1.84) |
| Median (IQR) | 3.8 (2.6–3.8) | 2.2 (1.5–3.1) | 2.1 (1.5–2.8) | 6.5 (2.5–18.4) |
| Range | 1.5–5.7 | 0.3–7.7 | 0.5–5.1 | 0.4–30.2 |
| Extrapolated outside range | 6 | 55 | 13 | 3 |
| OOR | 0 | 12 | 1 | 0 |
| Missing | 0 | 0 | 0 | 0 |
| IL-10 (pg/mL) |  |  |  |  |
| Geometric mean (CV) | 0.92 (0.32) | 0.74 (0.54) | 1.03 (5.38) | 1.64 (0.62) |
| Median (IQR) | 1.3 (0.9–1.3) | 1.3 (1.3–1.4) | 1.3 (1.3–1.3) | 2.7 (1.3–3.9) |
| Range | 0.4–1.3 | 0.7–3.9 | <LOD–9.56 | <LOD–9.62 |
| Extrapolated outside range | 4 | 16 | 0 | 2 |
| OOR | 9 | 58 | 14 | 3 |
| Missing | 0 | 0 | 0 | 0 |
| LIF (pg/mL) |  |  |  |  |
| Geometric mean (CV) | 6.57 (0.81) | 3.55 (1.76) | 4.61 (2.36) | 14.0 (0.57) |
| Median (IQR) | 4.0 (4.0–13.1) | 4.0 (3.4–12.7) | 4.0 (3.4–13.1) | 17.9 (12.7–20.8) |
| Range | <LOD–24.31 | 0.7–25.3 | 0.7–34.3 | 4.0–27.7 |
| Extrapolated outside range | 0 | 14 | 1 | 0 |
| OOR | 1 | 24 | 7 | 0 |
| Missing | 0 | 0 | 0 | 0 |
| SCF (pg/mL) |  |  |  |  |
| Geometric mean (CV) | 17.0 (0.54) | 9.52 (0.51) | 8.58 (0.77) | 9.62 (0.64) |
| Median (IQR) | 12.2 (8.9–14.9) | 10.7 (6.4–18.0) | 7.8 (5.6–10.0) | 14.4 (13.3–23.3) |
| Range | 3.2–22.6 | 0.7–44.2 | 0.7–25.5 | 2.0–30.9 |
| Extrapolated outside range | 1 | 13 | 4 | 2 |
| OOR | 0 | 1 | 0 | 0 |
| Missing | 0 | 0 | 0 | 0 |
| M-CSF (pg/mL) |  |  |  |  |
| Geometric mean (CV) | 18.0 (0.32) | 12.6 (0.34) | 11.6 (0.41) | 13.5 (0.40) |
| Median (IQR) | 11.7 (10.2–15.1) | 13.2 (9.4–20.5) | 9.6 (6.4–14.9) | 16.8 (12.2–26.0) |
| Range | 8.8–23.0 | 3.4–32.9 | 3.7–29.8 | 4.3–37.8 |
| Extrapolated outside range | 0 | 0 | 0 | 0 |
| OOR | 0 | 0 | 0 | 0 |
| Missing | 0 | 0 | 0 | 0 |
| TNF-α (pg/mL) |  |  |  |  |
| Geometric mean (CV) | 2.28 (0.60) | 2.08 (1.08) | 2.23 (0.69) | 2.81 (1.87) |
| Median (IQR) | 5.7 (1.8–5.7) | 5.7 (3.2–5.7) | 5.7 (5.7–5.7) | 5.4 (0.7–7.6) |
| Range | 1.8–6.9 | 0.6–30.2 | 1.8–11.7 | 0.6–16.2 |
| Extrapolated outside range | 6 | 42 | 4 | 7 |
| OOR | 7 | 45 | 12 | 2 |
| Missing | 0 | 0 | 0 | 0 |
| Descriptive data are presented as geometric mean and coefficients of variation (CV), medians with interquartile ranges (IQR), and ranges. Missing values, extrapolated values, and out-of-range (OOR) values are also provided.  Abbreviations: CI, confidence interval; CSF, cerebrospinal fluid; IFN, interferon; IL, interleukin; IQR, interquartile range; LIF, leukaemia inhibitory factor; LOD, limit of detection; M-CSF, macrophage colony-stimulating factor; MCP-1, monocyte chemoattractant protein-1; MMP, matrix metalloproteinases; OOR, out of range; SCF, stem cell factor. | | | | |

**Supplementary table 4.** Pairwise comparisons of blood-brain barrier disruption markers and inflammatory cytokines in women with normotensive pregnancies, pre-eclampsia without complications, pre-eclampsia with end-organ complications, and eclampsia, adjusted for plate differences.

|  | | **Fold change (95% CI) vs reference** | | |
| --- | --- | --- | --- | --- |
| **Biomarker** | **Reference** | **Pre-eclampsia end-organ without complications (n=88)** | **Pre-eclampsia with end-organ complications (n=17)** | **Eclampsia (n=11)** |
| Claudin-5 (pg/mL) | Normotensive | 0.91 (0.71, 1.18) p=0.47 | 0.75 (0.54, 1.05) p=0.087 | 3.05 (1.70, 5.46) p=0.001 |
|  | Pre-eclampsia without complications | — | 0.82 (0.63, 1.07) p=0.13 | 3.33 (1.91, 5.82) p<0.001 |
|  | Pre-eclampsia with end-organ complications | — | — | 4.08 (2.26, 7.35) p<0.001 |
| MMP-9 (pg/mL) | Normotensive | 0.99 (0.60, 1.62) p=0.95 | 0.75 (0.41, 1.39) p=0.35 | 1.78 (0.80, 3.94) p=0.15 |
|  | Pre-eclampsia without complications | — | 0.77 (0.48, 1.21) p=0.24 | 1.80 (0.88, 3.67) p=0.096 |
|  | Pre-eclampsia with end-organ complications | — | — | 2.35 (1.07, 5.15) p=0.034 |
| Albumin CSF/plasma ratio (x10^-3^) | Normotensive | 1.52 (1.23, 1.87) p<0.001 | 0.97 (0.70, 1.34) p=0.83 | 1.58 (0.89, 2.80) p=0.11 |
|  | Pre-eclampsia without complications | — | 0.64 (0.46, 0.88) p=0.009 | 1.04 (0.59, 1.84) p=0.88 |
|  | Pre-eclampsia with end-organ complications | — | — | 1.63 (0.89, 2.99) p=0.11 |
| IL-8 in CSF (pg/mL) | Normotensive | 1.12 (0.82, 1.53) p=0.45 | 1.31 (0.88, 1.96) p=0.18 | 8.77 (3.49, 22.05) p<0.001 |
|  | Pre-eclampsia without complications | — | 1.17 (0.83, 1.65) p=0.36 | 7.81 (3.18, 19.18) p<0.001 |
|  | Pre-eclampsia with end-organ complications | — | — | 6.69 (2.64, 16.93) p<0.001 |
| MCP-1 in CSF (pg/mL) | Normotensive | 0.90 (0.69, 1.18) p=0.44 | 0.74 (0.51, 1.06) p=0.097 | 3.16 (1.77, 5.64) p<0.001 |
|  | Pre-eclampsia without complications | — | 0.82 (0.60, 1.12) p=0.20 | 3.51 (2.03, 6.05) p<0.001 |
|  | Pre-eclampsia with end-organ complications | — | — | 4.29 (2.37, 7.75) p<0.001 |
| IL-6 in CSF (pg/mL) | Normotensive | 1.42 (0.68, 2.96) p=0.35 | 1.70 (0.70, 4.12) p=0.24 | 22.25 (8.06, 61.37) p<0.001 |
|  | Pre-eclampsia without complications | — | 1.20 (0.63, 2.28) p=0.57 | 15.71 (7.32, 33.70) p<0.001 |
|  | Pre-eclampsia with end-organ complications | — | — | 13.05 (5.11, 33.36) p<0.001 |
| IFN-γ in CSF (pg/mL) | Normotensive | 0.66 (0.42, 1.06) p=0.085 | 0.60 (0.34, 1.05) p=0.073 | 2.10 (1.11, 4.01) p=0.023 |
|  | Pre-eclampsia without complications | — | 0.90 (0.60, 1.36) p=0.63 | 3.17 (1.95, 5.16) p<0.001 |
|  | Pre-eclampsia with end-organ complications | — | — | 3.51 (1.94, 6.37) p<0.001 |
| IL-10 in CSF (pg/mL) | Normotensive | 0.81 (0.45, 1.46) p=0.49 | 1.12 (0.58, 2.17) p=0.73 | 1.79 (0.92, 3.48) p=0.087 |
|  | Pre-eclampsia without complications | — | 1.38 (0.87, 2.21) p=0.18 | 2.20 (1.43, 3.38) p<0.001 |
|  | Pre-eclampsia with end-organ complications | — | — | 1.59 (0.89, 2.84) p=0.12 |
| LIF in CSF (pg/mL) | Normotensive | 0.54 (0.27, 1.07) p=0.079 | 0.70 (0.30, 1.61) p=0.40 | 2.14 (0.84, 5.40) p=0.11 |
|  | Pre-eclampsia without complications | — | 1.30 (0.70, 2.41) p=0.41 | 3.96 (1.97, 7.94) p<0.001 |
|  | Pre-eclampsia with end-organ complications | — | — | 3.05 (1.27, 7.31) p=0.013 |
| SCF in CSF (pg/mL) | Normotensive | 0.56 (0.41, 0.77) p<0.001 | 0.50 (0.34, 0.74) p<0.001 | 0.57 (0.36, 0.88) p=0.012 |
|  | Pre-eclampsia without complications | — | 0.90 (0.68, 1.19) p=0.46 | 1.01 (0.72, 1.41) p=0.95 |
|  | Pre-eclampsia with end-organ complications | — | — | 1.12 (0.75, 1.69) p=0.58 |
| M-CSF in CSF (pg/mL) | Normotensive | 0.70 (0.57, 0.86) p=0.002 | 0.65 (0.49, 0.84) p=0.002 | 0.75 (0.55, 1.02) p=0.068 |
|  | Pre-eclampsia without complications | — | 0.92 (0.74, 1.14) p=0.45 | 1.07 (0.82, 1.40) p=0.57 |
|  | Pre-eclampsia with end-organ complications | — | — | 1.16 (0.85, 1.59) p=0.33 |
| TNF-α in CSF (pg/mL) | Normotensive | 0.91 (0.46, 1.80) p=0.79 | 0.98 (0.42, 2.27) p=0.96 | 1.23 (0.52, 2.95) p=0.64 |
|  | Pre-eclampsia without complications | — | 1.07 (0.57, 2.02) p=0.82 | 1.35 (0.74, 2.48) p=0.33 |
|  | Pre-eclampsia with end-organ complications | — | — | 1.26 (0.55, 2.88) p=0.59 |
| Statistical analyses of IFN-γ, LIF, SCF and IL-10 in CSF were performed using log-normal Tobit regression, accounting for censoring of values below the detection limit. Analyses of claudin-5, MMP-9, MCP-1, M-CSF in CSF were performed using Welch’s analysis of covariance (ANCOVA) on log-transformed variables to account for unequal variances across groups. Results are presented as fold changes vs. reference with 95% confidence intervals, adjusting for plate effects. Abbreviations: CI, confidence interval; CSF, cerebrospinal fluid; IFN, interferon; IL, interleukin; LIF, leukaemia inhibitory factor; M-CSF, macrophage colony-stimulating factor; MCP-1, monocyte chemoattractant protein-1; MMP, matrix metalloproteinases; SCF, stem cell factor; TNF, tumour necrosis factor. | | | | |

**Supplementary table 5.** Descriptive data of inflammatory cytokines, chemokines and growth factors in CSF and plasma in women with normotensive pregnancies, pre-eclampsia without complications, pre-eclampsia with end-organ complications, and eclampsia, highlighting markers that were not detectable across all study groups.

|  | **Normotensive**  **(n=13)** | **Pre-eclampsia without complications**  **(n=88)** | **Pre-eclampsia with end-organ complications (n=17)** | **Eclampsia**  **(n=11)** |
| --- | --- | --- | --- | --- |
| CSF |  |  |  |  |
| GRO-α | 0 (0%) | 1 (1.2%) | 0 (0%) | 5 (45%) |
| IFN-α2 | 0 (0%) | 0 (0%) | 0 (0%) | 0 (0%) |
| IL-15 | 2 (15%) | 9 (10%) | 1 (5.3%) | 0 (0%) |
| VEGF | 0 (0%) | 4 (4.7%) | 2 (11%) | 0 (0%) |
| β-NGF | 0 (0%) | 32 (37%) | 1 (5.3%) | 6 (55%) |
| Plasma |  |  |  |  |
| IFN-γ | 0 (0%) | 27 (31%) | 2 (11%) | 6 (55%) |
| IL-3 | 0 (0%) | 5 (5.8%) | 0 (0%) | 0 (0%) |
| IL-5 | 0 (0%) | 1 (1.2%) | 0 (0%) | 0 (0%) |
| Abbreviations**:** β-NGF, beta nerve growth factor; GRO-α, melanoma growth stimulating activity alpha; IFN, interferon; IL, interleukin; VEGF, vascular endothelial growth factor. | | | | |

**Supplementary table 6.** Correlations between inflammatory cytokines in CSF and plasma.

| **Biomarker** | **Correlation coefficient (95% CI)** |
| --- | --- |
| IL-6 | 0.16 (-0.01, 0.33)   p=0.072 |
| IL-8 | 0.05 (-0.15, 0.23)   p=0.64 |
| TNF-α | 0.26 (-0.06, 0.54)   p=0.12 |
| Correlation coefficients for censored data (TNF-α and IL-6) were estimated by using the clikcorr R package with log-Gaussian profile likelihood estimation. Abbreviations**:** CI, confidence interval; IL, interleukin; TNF, tumour necrosis factor. | |

**Supplementary table 7.** Comparison of women with eclampsia included in the current study to women with eclampsia recruited to the PROVE biobank as reported in previous publications.^22,60,63^

|  | Eclampsia  (n=11) | Eclampsia, stroke or blindness  Bergman et al., Cells, 2021^21^  (n=86) | Eclampsia  Bergman et al., AJOG, 2021^62^  (n=16) | Eclampsia  Bergman et al, AJOG, 2024^59^  (n=49) |
| --- | --- | --- | --- | --- |
| Age (years), mean (SD) | 26·3 (6·9) | 22·8 (6·1) | 23·5 (5·9) | 22·4 (5·6) |
| Body mass index (kg/m^2^), mean (SD) | 29·2 (5·0) | 26·4 (8·1) | 25·2 (3·4) | 24·8 (5·6) |
| Nulliparous, n (%) | 6 (55%) | 60 (70%) | 10 (63%) | 36 (74%) |
| HIV, n (%) | 2 (18%) | 10 (12%) | 1 (6%) | 6 (12%) |
| Smoking during pregnancy, n (%) | 1 (9%) | — | 4 (27%) | 9 (18%) |
| Alcohol use during pregnancy, n (%) | 0 (0%) | — | 3 (20%) | 7 (14%) |
| Chronic hypertension, n (%) | 0 (0%) | 7(8%) | 1 (6%) | 2 (4%) |
| Pulmonary oedema, n (%) | 3 (27%) | 5 (6%) | 1 (6%) | 1 (2%) |
| HELLP syndrome, n (%) | 1 (9%) | 19 (22%) | 1 (6%) | 14 (29%) |
| Severe renal impairment (creatinine >120 µmol/L), n (%) | 0 | 16 (19%) | 1 (6%) | 10 (20%) |
| Placental abruption, n (%) | 0 | 5 (6%) | — | 3 (6%) |
| Recurrent eclampsia, n (%) | 3 (27%) | 26 (30%) | — | 17 (35%) |
| Elective or non-urgent caesarean section, n (%) | 1 (9%) | 1 (1%) | 0 (0%) | 1 (2%) |
| Emergency caesarean section, n (%) | 10 (91%) | 60 (70%) | 13 (81%) | 31 (63%) |
| Vaginal delivery, n (%) | 0 | 1 (1%) | 3 (19%) | 17 (35%) |
| Gestation at delivery (weeks+days), median (IQR) or mean (SD) | 37+2 (34+1–39+4) | 33·4 (4·3) | 34·3 (4·3) | 34+4 (24+2–40+5) |
| Liveborn infant, n (%) | 11 (100%) | - | 16 (100%) | 41 (84%) |
| Birthweight (kg), mean (SD) | 2·5 (0·7) | 2·1 (0·9) | 2·1 (0·8) | 2·1 (0·9) |
| Numeric variables are presented as mean with standard deviation (SD) or median with interquartile range (IQR), while categorical variables are reported as counts and percentages.  Missing data are as follows (by group: normotensive, pre-eclampsia without end-organ complications, pre-eclampsia with end-organ complications, and eclampsia): body mass index – 3/3/1/1; smoking status – 0/0/1/0; chronic hypertension – 0/1/0/0; emergency caesarean section – 0/1/1/0; and birthweight – 0/1/0/0. Abbreviations: HELLP, haemolysis, elevated liver enzymes, low platelet count; HIV, human immunodeficiency virus; IQR, interquartile range; SD, standard deviation. | | | | |

**Supplementary figure 1.**

 Correlations between CSF concentrations of matrix metalloproteinase (MMP)-9 vs claudin-5 (**a**), and CSF/plasma albumin ratio vs claudin-5 (**b**) and CSF/plasma albumin ratio vs MMP-9 (**c**). Pearson correlation coefficient (95% CI) is presented on log-transformed values. For censored data correlation coefficients were estimated by using log-Gaussian profile likelihood using the clikcorr R package

**Supplementary figure 2.**

 Correlations between inflammatory cytokines interleukin-8 (IL-8) vs interleukin-6 (IL-6) (**a**), tumour necrosis factor alpha (TNF-α) vs IL-6 (**b**) and TNF-α vs IL-8 (**c**) in CSF. Pearson correlation coefficient (95% CI) is presented on log-transformed values. For censored data correlation coefficients were estimated by using log-Gaussian profile likelihood using the clikcorr R package.

**Supplementary figure 3.**

 Correlations between inflammatory cytokines interleukin-6 (IL-6) (**a**), interleukin-8 (IL-8) (**b**) and tumour necrosis factor alpha (TNF-α) (**c**) in CSF vs plasma. Pearson correlation coefficient (95% CI) is presented on log-transformed values. For censored data (TNF-α and IL-6) correlation coefficients were estimated by using log-Gaussian profile likelihood using the clikcorr R package.

**Supplementary checklist 1:** STROBE Statement—Checklist of items that should be included in reports of ***cross-sectional studies***.

|  | Item No | Recommendation | Page No |
| --- | --- | --- | --- |
| **Title and abstract** | 1 | (*a*) Indicate the study’s design with a commonly used term in the title or the abstract | 3 |
|  |  | (*b*) Provide in the abstract an informative and balanced summary of what was done and what was found | 3-4 |
| Introduction | | | |
| Background/rationale | 2 | Explain the scientific background and rationale for the investigation being reported | 7 |
| Objectives | 3 | State specific objectives, including any prespecified hypotheses | 7 |
| Methods | | | |
| Study design | 4 | Present key elements of study design early in the paper | 8 |
| Setting | 5 | Describe the setting, locations, and relevant dates, including periods of recruitment, exposure, follow-up, and data collection | 8-10 |
| Participants | 6 | (*a*) Give the eligibility criteria, and the sources and methods of selection of participants | 8 |
| Variables | 7 | Clearly define all outcomes, exposures, predictors, potential confounders, and effect modifiers. Give diagnostic criteria, if applicable | 9-10 |
| Data sources/ measurement | 8* | For each variable of interest, give sources of data and details of methods of assessment (measurement). Describe comparability of assessment methods if there is more than one group | 10 |
| Bias | 9 | Describe any efforts to address potential sources of bias | 11,21 |
| Study size | 10 | Explain how the study size was arrived at | 12, 13, Figure 1 |
| Quantitative variables | 11 | Explain how quantitative variables were handled in the analyses. If applicable, describe which groupings were chosen and why | 11 |
| Statistical methods | 12 | (*a*) Describe all statistical methods, including those used to control for confounding | 11 |
|  |  | (*b*) Describe any methods used to examine subgroups and interactions | 11 |
|  |  | (*c*) Explain how missing data were addressed | 11 |
|  |  | (*d*) If applicable, describe analytical methods taking account of sampling strategy | NA |
|  |  | (*e*) Describe any sensitivity analyses | NA |
| Results | | | |
| Participants | 13* | (a) Report numbers of individuals at each stage of study—eg numbers potentially eligible, examined for eligibility, confirmed eligible, included in the study, completing follow-up, and analysed | 13, Figure 1 |
|  |  | (b) Give reasons for non-participation at each stage | Figure 1 |
|  |  | (c) Consider use of a flow diagram | Figure 1 |
| Descriptive data | 14* | (a) Give characteristics of study participants (eg demographic, clinical, social) and information on exposures and potential confounders | 13, Table 1 |
|  |  | (b) Indicate number of participants with missing data for each variable of interest | Figure 1, Table 1, Supp. Table 3 |
| Outcome data | 15* | Report numbers of outcome events or summary measures | Table 1, Supp. Table 3,4 and 5 |
| Main results | 16 | (*a*) Give unadjusted estimates and, if applicable, confounder-adjusted estimates and their precision (eg, 95% confidence interval). Make clear which confounders were adjusted for and why they were included | Supp. Table 4 and 6, Table 2, 3, 4 and 5 |
|  |  | (*b*) Report category boundaries when continuous variables were categorized | NA |
|  |  | (*c*) If relevant, consider translating estimates of relative risk into absolute risk for a meaningful time period | NA |
| Other analyses | 17 | Report other analyses done—eg analyses of subgroups and interactions, and sensitivity analyses | 16-17, Table 4 and 5, |
| Discussion | | | |
| Key results | 18 | Summarise key results with reference to study objectives | 18 |
| Limitations | 19 | Discuss limitations of the study, taking into account sources of potential bias or imprecision. Discuss both direction and magnitude of any potential bias | 21 |
| Interpretation | 20 | Give a cautious overall interpretation of results considering objectives, limitations, multiplicity of analyses, results from similar studies, and other relevant evidence | 18-21 |
| Generalisability | 21 | Discuss the generalisability (external validity) of the study results | 21 |
| Other information | | | |
| Funding | 22 | Give the source of funding and the role of the funders for the present study and, if applicable, for the original study on which the present article is based | 23 |

*Give information separately for exposed and unexposed groups.

**Note:** An Explanation and Elaboration article discusses each checklist item and gives methodological background and published examples of transparent reporting. The STROBE checklist is best used in conjunction with this article (freely available on the Web sites of PLoS Medicine at http://www.plosmedicine.org/, Annals of Internal Medicine at http://www.annals.org/, and Epidemiology at http://www.epidem.com/). Information on the STROBE Initiative is available at www.strobe-statement.org.
